# Redirecting anti-Vaccinia virus T cell immunity for cancer treatment by AAV-mediated delivery of the VV B8R gene

**DOI:** 10.1016/j.omto.2022.04.008

**Published:** 2022-04-25

**Authors:** Dujuan Cao, Qianqian Song, Junqi Li, Louisa S. Chard Dunmall, Yuanyuan Jiang, Bin Qin, Jianyao Wang, Haoran Guo, Zhenguo Cheng, Zhimin Wang, Nicholas R. Lemoine, Shuangshuang Lu, Yaohe Wang

**Affiliations:** 1 National Center for International Research in Cell and Gene Therapy, Sino-British Research Centre for Molecular Oncology, School of Basic Medical Sciences, Academy of Medical Sciences, Zhengzhou University, Zhengzhou, China; 2 Centre for Cancer Biomarkers & Biotherapeutics, Barts Cancer Institute, Queen Mary University of London, London, UK

**Keywords:** Adeno-associated virus, B8R, Vaccinia virus, Virus-specific memory T cells, Tumor regression

## Abstract

Immunotherapies, such as immune checkpoint inhibitors (ICIs) and chimeric antigen receptor-T (CAR-T) cells, are only efficient in a small proportion of tumor patients. One of the major reasons for this is the lack of immune cell infiltration and activation in the tumor microenvironment (TME). Recent research reported that abundant bystander CD8^+^ T cells targeting viral antigens exist in tumor infiltrates and that virus-specific memory T cells could be recalled to kill tumor cells. Therefore, virus-specific memory T cells may be effective candidates for tumor immunotherapy. In this study, we established subcutaneous tumor mice models that were pre-immunized with Vaccinia virus (VV) and confirmed that tumor cells with ectopic expression of the viral B8R protein could be recognized and killed by memory T cells. To create a therapeutic delivery system, we designed a recombinant adeno-associated virus (rAAV) with a modified tumor-specific promoter and used it to deliver VV B8R to tumor cells. We observed that rAAV gene therapy can retard tumor growth in VV pre-immunized mice. In summary, our study demonstrates that rAAV containing a tumor-specific promoter to restrict VV B8R gene expression to tumor cells is a potential therapeutic agent for cancer treatment in VV pre-immunized or VV-treated mice bearing tumors.

## Graphical abstract

**Figure:**
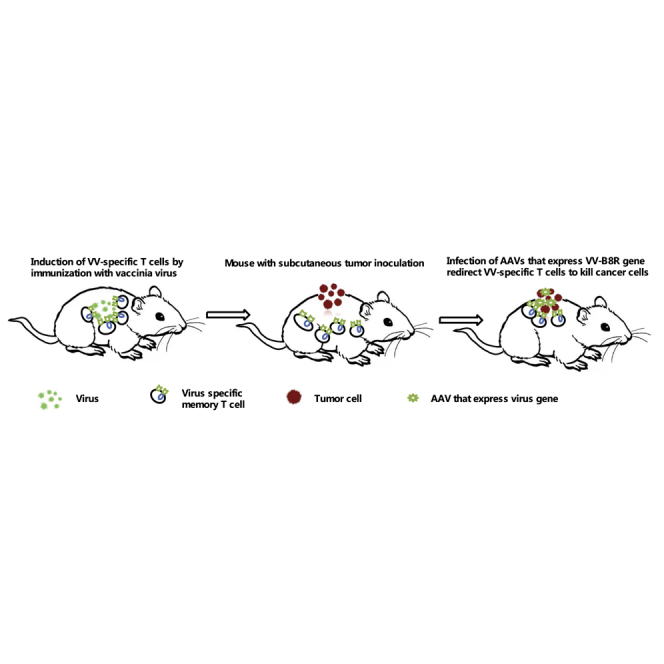

## Teaser

Authors of this manuscript constructed a recombinant adeno-associated virus (rAAV) with a new tumor-specific promoter for ectopic expression of B8R gene of Vaccinia virus (VV) in tumor tissues. In VV pre-immunized mice, treatment with rAAV-B8R recruited anti-B8R T cells to the tumor, demonstrating significant antitumor efficacy.

## Introduction

Cancer remains a major public health problem worldwide and a leading cause of death despite numerous efforts to address prevention and control.^1^ While surgery, radiotherapy, and chemotherapy remain the standard of care for most cancers, gene therapy and immunotherapy are emerging as powerful new therapeutics and have achieved extraordinary outcomes in the past 3 decades.^2^

During gene therapy protocols, functional genes are delivered into cells of a patient to correct a genetic error or provide a new function to the cell. Gene therapy was initially developed to treat inherited genetic diseases, but more recently, a role has emerged for treatment of cancer.^3^ More than two-thirds of the clinical and preclinical gene therapy approaches have now been applied to cancer. Based on targets, cancer gene therapy can be classified into two types. Molecular targets consist of tumor-suppressor gene therapy, down-regulation of oncogenes, and manipulating drug effects. Immunological targets include passive and active immune modulation.^4^ Passive immune-modulation strategies involve the transfection of immune effector cells with cytokine genes to enhance their cytotoxicity to tumor cells. Active immune-modulation strategies involve the genetic modification of tumor cells by transfection of cytokine genes or tumor antigens to more potently activate immune responses.^5^^,^^6^

Targeting T cell immunotherapies, such as immune checkpoint inhibitors (ICIs) or chimeric antigen receptor-T (CAR-T) therapy, have emerged as an important treatment modality and have shown promising results in some patient groups. However, limitations to these treatments exist. CAR-T therapies for solid tumors are hampered by toxicity, a lack of tumor antigen candidates, and an immune-suppressive tumor microenvironment (TME) that prevents infiltration and effective activation of CAR-T cells.^7^ ICI strategies also fail due to a lack of T cell infiltration and activation.^8^ Many patients present with “cold tumors” that harbor an immunosuppressive microenvironment not conducive to T cell activation, and these patients will fail to respond effectively to ICIs.^9^

Recent research has suggested that there are abundant bystander CD8^+^ T cells in tumor infiltrates and most of these T cells target viruses.^10^ Theoretically, these virus-specific T cells are great candidates for *in situ* tumor treatment if tumor cells can present viral antigens. Several research groups have developed different methods of presenting viral antigens in tumor cells to activate the virus-specific T cells to target tumor cells, and these methods have shown significant efficacy for tumor treatment.^11^^,^^12^ One method reported the use of antibody-targeted, pathogen-derived peptides (ATPP) in which tumor-cell-specific antibodies are used to deliver immunogenic T cell epitopes into the major histocompatibility complex (MHC) class I molecules on the tumor’s cell surface.^11^ However, this method has disadvantages: ATPP cannot target all tumor cells because there are rare tumor-cell-membrane-specific antigens and ATPP may be taken up by normal cells, leading to off-target effects. Another method involves repeated intra-tumoral (i.t.) injection of viral peptides to activate virus-specific memory T cells to target tumor cells;^12^ however, the feasibility and safety of repeated i.t. peptide delivery in the clinical setting needs further investigation, and viral peptides have to be matched to the patient histocompatibility leukocyte antigen (HLA) type.

Here, we chose to activate Lister strain Vaccinia virus (VV)-specific memory T cells as a mechanism of tumor elimination because the Lister strain VV was used extensively in the UK, Africa, Asia, and Oceania during the smallpox eradication campaign.^13^^,^^14^ The smallpox vaccine induces virus-specific memory T cells that can last for decades, and these memory T cells can self-renew without stimulation by virus antigens.^15^ We found that ectopic expression of the most immunogenic gene of VV (B8R) in tumor cells can antagonize tumor growth in VV pre-immunized mice.

In order to conquer the disadvantages of previous methods for virus antigen presentation in tumor cells, we used recombinant adeno-associated virus (rAAV) to deliver the viral B8R gene to tumor cells. AAV is a single-stranded DNA virus that can infect both dividing and non-dividing cells.^16^ rAAV has been extensively explored as a gene therapy vector, as it encompasses a number of features that suit this objective, including non-pathogenicity, broad host tissue range, and long-term transgene expression.^17^ However, the wide host range of AAV can increase side effects of tumor treatment.^18^ One way to address this problem is to use a tumor-specific promoter in the AAV vector design. Telomerase reverse transcriptase (TERT), which is required for elongation of telomeres, is highly expressed and activated in more than 90% of tumor cells, but not in normal healthy tissues.^19^^,^^20^ Many factors were reported that can influence the activity of TERT promoter, such as a c-Myc binding site,^21^ point mutation in TERT promoter,^22^ and presence of an antioxidative response element (ARE).^23^ We combined all these features to create a potent promoter that is tumor-cell-specific (termed the ATM promoter). We constructed a rAAV with the ATM promoter to express the VV B8R gene specifically in tumor cells and activate VV-specific memory T cells to antagonize cancer growth and eliminate tumors.

## Results

### Vaccinia virus pre-immunization enhances the antitumor efficacy of oncolytic VV

**Figure 1:**
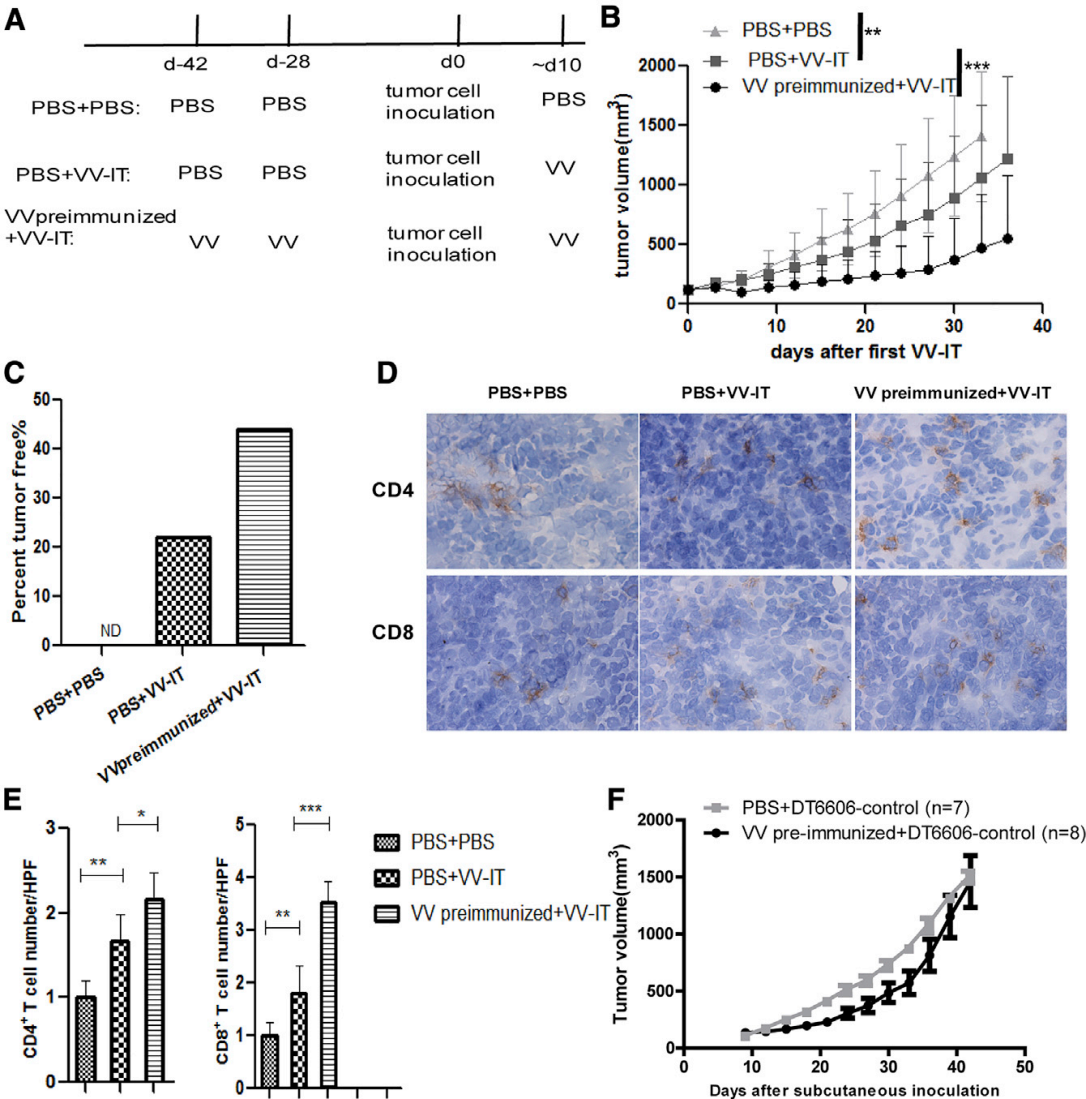
Vaccinia virus pre-immunization enhances the antitumor efficacy of oncolytic VV (A) Schematic of the experimental setup. For pre-immunization, C57/Bl6 mice were immunized using 100 μL PBS or VV at an MOI of 5 × 10^6^ PFUs/mouse through i.m. injection twice with a 2-week interval. Four weeks after VV or mock immunization, 1 × 10^6^ DT6606 cells were inoculated into the flanks of the mice. About 10 days later, once the volume of the tumor reached 100 mm^3^, 100 μL PBS or 1 × 10^8^ PFUs VV/mouse were administered through i.t. injection daily for 5 consecutive days. (B) Tumor growth curve is shown (n = 9/group). Mice were treated as described in (A). Tumor growth was monitored twice per week, and mean tumor volume ± SEM was shown. (C) The percentage of tumor-free mice at the experimental endpoint in indicated groups is shown. Mice were treated as described in (A). (D) IHC analysis of CD4^+^ and CD8^+^ T cells in tumor tissues of treated mice 7 days after VV i.t. injection is shown. Magnification 400× is shown. Mice were treated as described in (A). (E) CD4^+^ T cells and CD8^+^ T cells were quantified in each group (n = 3/group). Ten high-power fields (HPFs) were counted in each mouse. The average positive cell number/HPF was calculated, and the mean ± SEM is shown. (F) Mice were pre-immunized by PBS or VV as described in (A). Four weeks after pre-immunization, 1 × 10^6^ DT6606-control cells (DT6606 cell infected by empty lentivirus vector) were inoculated into the flanks of the mice. Tumor volume was monitored every 3 days and tumor growth was recorded. ∗p < 0.05, ∗∗p < 0.01, and ∗∗∗p < 0.001.

We used Vaccinia virus VVLΔTKΔN1L-RFP (referred to from now on as VV) as previously reported^24^ for both pre-immunization and i.t. therapeutic injection. In order to visualize whether VV-specific memory T cells can elicit an antitumor effect, we pre-immunized mice by intramuscular (i.m.) injection of VV or PBS and then inoculated the pancreatic cancer cell line DT6606 subcutaneously 4 weeks after immunization (Figure 1A). After the tumor volume reached 100 mm^3^, we treated mice by i.t. injection of VV and found tumor growth rate was reduced in the VV pre-immunized group compared with the PBS pre-immunized group (Figure 1B), and VV pre-immunization resulted in a higher percentage of tumor-free mice after therapeutic intervention with VV (44% versus 22%; Figure 1C). The levels of tumor-infiltrated CD4- and CD8-positive T cells in the VV pre-immunized group were higher compared with the PBS pre-immunized group (Figures 1D and 1E). VV pre-immunization did not cause non-specific effects to VV-untreated tumors. We compared DT6606 tumor cell growth rate in VV pre-immunized mice and PBS immunized mice and found no significant difference (Figure 1F). These results suggested that VV-specific memory T cells can be activated quickly and recruited to the site of VV infection to regress tumor growth.

Although VV i.t. injection regressed tumor growth rate, the effect was modest after 20 days of VV injection (Figure 1B). We speculate that the reason for the modest effect may be due to the low infection efficiency of VV in tumor cells, coupled with immune-mediated clearance of VV in tumors after 20 days (Figure S1).

### Ectopic expression of VV B8R protein can delay tumor growth in VV pre-immunized mice

**Figure 2:**
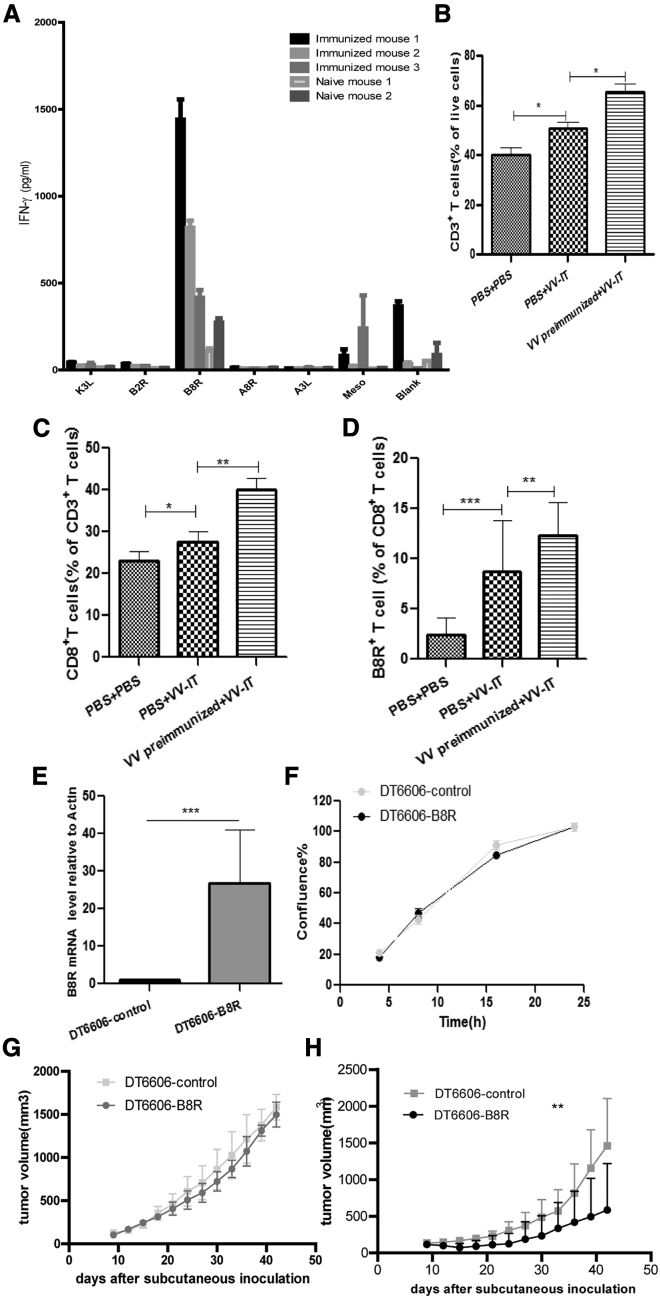
Ectopic expression of the VV-B8R protein can delay tumor growth in VV pre-immunized mice (A) ELISA detection of IFN-γ release in splenocytes of VV immunized mice and naive mice that were stimulated by different VV peptides. Mice were immunized with VV at an MOI of 5 × 10^6^ PFUs/mouse (immunized group) or left unimmunized (naive group) and sacrificed 2 weeks later. Splenocytes were co-cultured with indicated peptides for 72 h, and IFN-γ level in the supernatants was detected by ELISA. The ELISA assay was repeated three times, and the mean ± SEM was shown. (B–D) The proportion of CD3^+^ T cells (B), CD8^+^ T cells (C), and B8R^+^ T cells (D) in tumors of indicated mice groups is shown; n = 3/group. Mean ± SEM is shown. Mice were treated as described in Figure 1A. (E) qPCR detection of B8R mRNA levels in DT6606-control and DT6606-B8R cell lines is shown. (F) Cell growth rate of DT6606-control and DT6606-B8R cell lines *in vitro* is shown. We seeded 5 × 10^4^ cells in each well in a 96-well plate, and cell growth was monitored by IncuCyte Zoom. The percentage of confluence is plotted. (G) Tumor growth rate of DT6606-control and DT6606-B8R cell lines in C57/Bl6 mice is shown; n = 8/group. We inoculated 1 × 10^6^ tumor cells into 5- to 6-week-old C57/Bl6 mice subcutaneously, and tumor growth was monitored twice per week. Mean tumor growth ± SEM is shown. (H) Tumor growth rate of DT6606-control and DT6606-B8R cell lines in VV pre-immunized mice is shown; n = 8/group. For pre-immunization, 3- to 4-week-old C57/Bl6 mice were infected by VV through i.m. injection at an MOI of 5 × 10^6^ PFUs/mouse twice with a 2-week interval as previously described. Four weeks after VV immunization, 1 × 10^6^ tumor cells were inoculated to the back of these mice, and tumor growth was monitored twice per week. Mean tumor growth ± SEM is shown. ∗p < 0.05, ∗∗p < 0.01, and ∗∗∗p < 0.001 between the indicated groups of this figure.

In order to identify immunogenic VV genes that can be used for tumor gene therapy, we immunized mice using VV and then stimulated splenocytes from these mice *ex vivo* with previously reported immunogenic peptides derived from K3L, B2R, B8R, A8R, and A3L of VV.^25^ These peptides were determined to be immunogenic in the Western Reserve (WR) VV strain. Both WR and Lister VV strains can be used for tumor therapy, but they have different immunogenicity.^26^ Through comparisons of the genomes of WR with Lister strain viruses, we found they were also present in the Lister strain with the same predicted sequence and function. Through interferon (IFN)-γ ELISA assay, we identified that the B8R peptide was the most immunogenic peptide of the Lister VV strain (Figure 2A). i.t. injection of VV (daily for 5 days) enhanced the CD3^+^ T cell, CD8^+^ T cell, and B8R-specific CD8^+^ T cell infiltration into the tumor, and this effect was greater in pre-immunized compared with naive mice (Figures 2B–2D). These results suggested that ectopic expression of B8R in tumor cells may contribute to the enhanced antitumor efficacy of VV in VV pre-immunized mice.

In order to detect whether B8R expression in tumor cells can trigger VV-specific memory T cells to eliminate tumor cells, we constructed a DT6606 cell line that stably expressed VV-B8R through lentivirus infection (Figure 2E). Compared with control lentivirus-infected DT6606 cells (DT6606-control), overexpression of B8R did not influence the growth rate of DT6606 cells (DT6606-B8R) either *in vitro* (Figure 2F) or *in vivo* (Figure 2G). We pre-immunized mice with VV, and 4 weeks later, we inoculated DT6606-control and DT6606-B8R cells subcutaneously. After pre-immunization, the tumor growth rate of DT6606-B8R cells was significantly slower than that of DT6606-control cells (Figure 2H). These results indicated that ectopic expression of B8R in tumor cells can regress tumor growth in VV pre-immunized mice.

Of note, splenocytes from PBS pre-immunized mice + VV-i.t. treatments and splenocytes from VV-pre-immunized + VV-i.t. treatment were able to kill B8R-negative DT6606 cells *ex vivo* through stimulation using a B8R peptide or heat-inactivated VV (Figures S2B and S2C). This cytotoxicity is tumor cell specific, as the splenocytes from VV pre-immunized + VV-i.t. treatment could only kill DT6606 cells and not unrelated KYSE180 cells (Figure S2D). This demonstrates that our vaccination strategy triggered cytotoxicity toward cells expressing epitopes other than B8R, likely through antigen spreading.

### rAAV with a robust tumor-cell-specific promoter can be used to deliver B8R for expression in tumor cells *in vivo*

**Figure 3:**
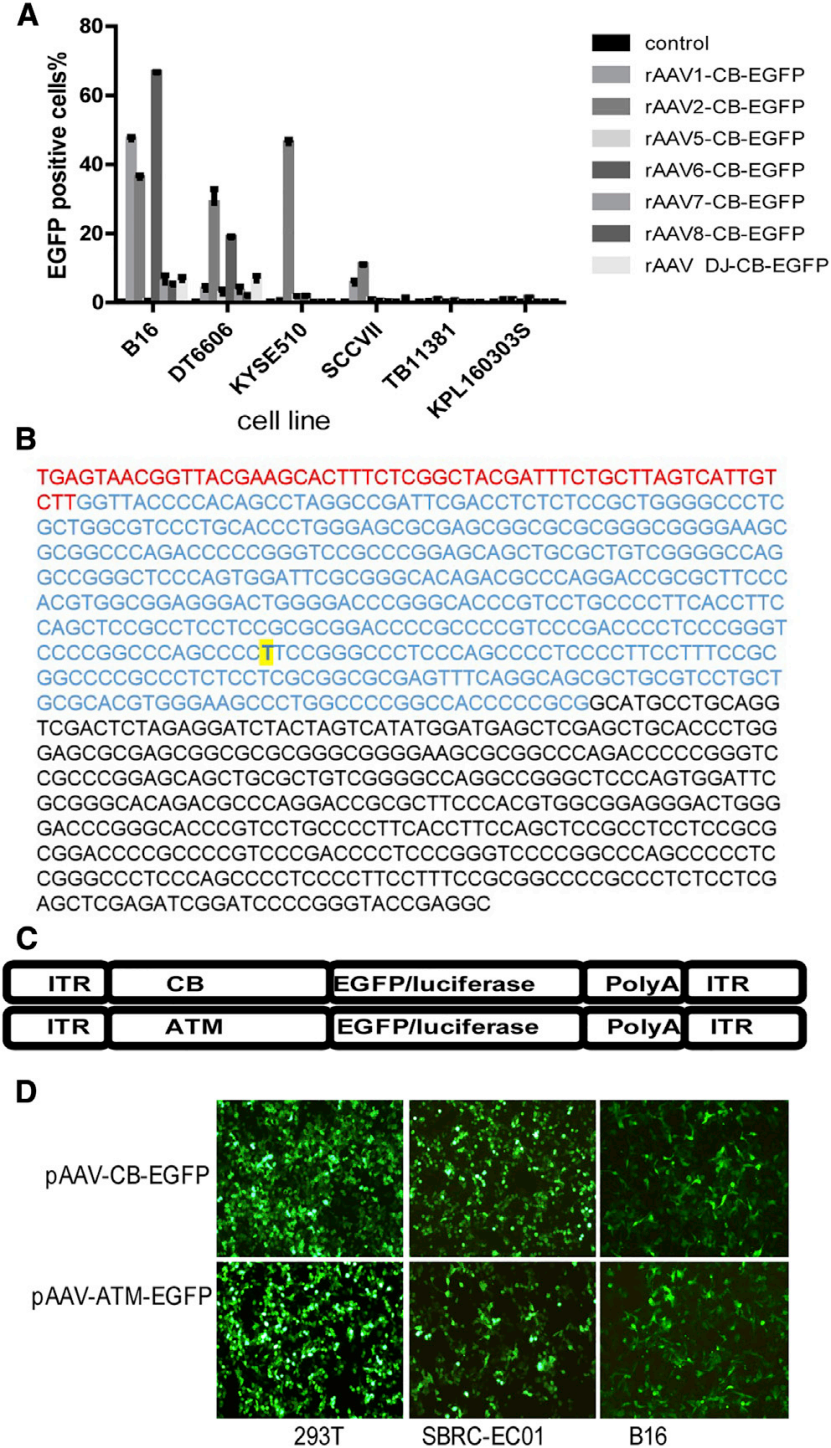
Construction of a rAAV vector with a novel tumor-cell-specific promoter (A) Infection efficiency of different serotypes of AAVs to different tumor cell lines, indicated by the proportion of EGFP-positive cells. B16: mouse melanoma cell line; DT6606 and TB11381: mouse pancreatic cancer cell line; KYSE510: human esophageal squamous cell line; SCCVII: mouse squamous cell carcinoma cell line; KPL160303S: mouse lung cancer cell line. AAV infection MOI = 10^5^ vg/cell. (B) The DNA sequence of the ATM promoter is shown. Red, ARE sequence; blue, hTERT promoter sequence from position −441 to −1, yellow-highlighted base indicates the C to T mutation at −124 site, and black, c-Myc binding sequence. (C) Schematic of the rAAV vector inserts used in this manuscript is shown. ITR, inverted terminal repeat; CB, chicken β-actin promoter plus cytomegalovirus enhancer; ATM, promoter composed of ARE, hTERT promoter, and c-Myc binding site. (D) EGFP expression in indicated cell lines that were transfected by corresponding AAV plasmids with different promoters is shown. Magnification 200× is shown.

Having demonstrated the potential of B8R as a therapeutic molecule for cancer in VV pre-immunized mice, we next sought to create an AAV-based vector to deliver the B8R gene for expression in tumor cells. Previous research has reported that different AAV serotypes show different levels of infectivity to various tissues.^27^ Analysis of different AAV serotype infectivity in a panel of tumor cells indicated that rAAV6 could infect B16 cells most efficiently, whereas rAAV2 could infect DT6606 cells most efficiently compared with other AAV serotypes (Figure 3A), suggesting the rational selection of the appropriate serotype of rAAV to target specific tumor cells.

In order to express the B8R protein only in tumor cells, we synthesized a new telomerase-based, tumor-cell-specific promoter, termed ATM, based on literature regarding sequence alterations that enhance promoter activity.^22^^,^^23^ The ATM promoter was composed of an antioxidant response element (ARE), a human telomerase reverse transcriptase (hTERT) promoter with C to T mutation at nucleotide −124 site and a c-Myc binding sequence (Figure 3B). We constructed a rAAV vector containing the ATM promoter (Figure 3C) and compared its expression intensity with the broad-spectrum CB (chicken β-actin promoter plus cytomegalovirus enhancer) promoter in immortalized 293T cells, the human esophageal squamous carcinoma cell line SBRC-EC01, and the mouse melanoma cell line B16 through plasmid transfection. We found the expression intensity was similar between the two plasmids with different promoters in these cell lines (Figure 3D).

**Figure 4:**
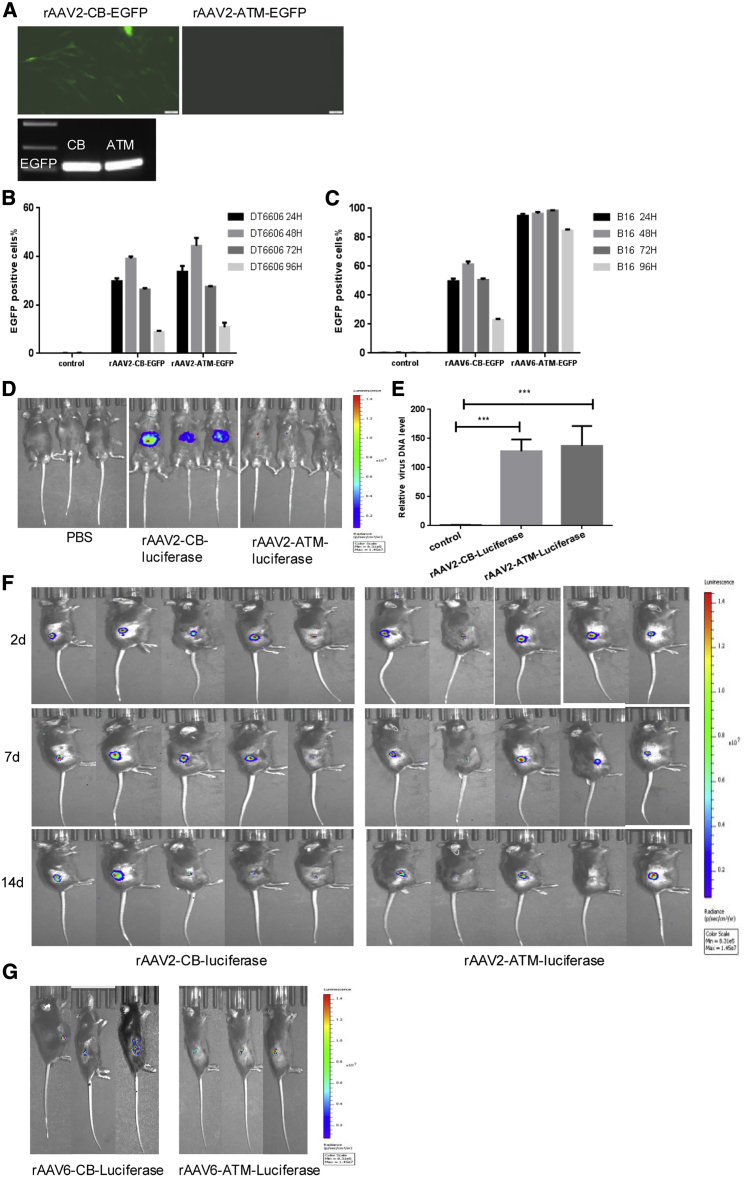
The ATM promoter is a strict tumor-specific promoter (A) Upper: EGFP expression after rAAV infection of human hepatocytes derived from iPSCs. Cells were infected with indicated rAAV at an MOI of 1 × 10^5^ vg/cell. Lower: cell genome PCR of EGFP to detect successful infection by indicated virus is shown. (B) Proportion of EGFP-positive DT6606 cells that were infected by indicated rAAVs at an MOI of 1 × 10^5^ vgs/cell at different time points (24 h, 48 h, 72 h, and 96 h) after virus infection is shown. n = 3/group. Mean ± SEM is shown. (C) Proportion of EGFP-positive B16 cells that were infected by indicated rAAVs at a MOI of 1 × 10^5^ vgs/cell at different time points is shown. n = 3/group. Mean ± SEM is shown. (D) *In vivo* imaging to detect luciferase signals in mice 7 days after infection by indicated rAAV is shown. Mice were infected by rAAV at an MOI of 1 × 10^11^ vgs/mouse through tail vein injection (n = 3/group). (E) PCR detection of the virus DNA level in livers of mice infected by indicated rAAV in (D) is shown. n = 3/group. Mean ± SEM is shown. (F) *In vivo* imaging to detect luciferase signals in mice with DT6606 subcutaneous tumors at different time points (2 days, 7 days, and 14 days) after i.t. injection of indicated rAAVs is shown. Mice were infected by rAAV at an MOI of 1 × 10^11^ vgs/mouse through i.t. injection (n = 5/group). (G) *In vivo* imaging to detect luciferase signals in mice with B16 subcutaneous tumors 14 days after i.t. injection of indicated rAAVs is shown. Mice were infected by rAAV at an MOI of 1 × 10^11^ vg/mouse through i.t. injection. ∗∗∗p < 0.001 between the indicated groups of this figure.

We next packaged different serotypes of rAAV-ATM-EGFP/luciferase and rAAV-CB-EGFP/luciferase to detect the tumor specificity of the ATM promoter both *in vitro* and *in vivo*. For *in vitro* detection, we used rAAV2-ATM/CB-EGFP to infect human hepatocytes differentiated from induced pluripotent stem cells (iPSCs) and found that only the CB promoter triggered EGFP expression in hepatocytes, although cell-genome PCR analysis showed that both rAAVs infected hepatocytes efficiently (Figure 4A). We then used the appropriate serotypes of rAAVs (as determined in Figure 3A) to infect DT6606 and B16 cells and found that both the CB and ATM promoters could trigger EGFP expression in these tumor cell lines. As expected, the proportion of EGFP-positive cells decreased along with cell division (Figures 4B and 4C). To investigate tumor specificity *in vivo*, we packaged different rAAV viruses as indicated in the Figures 4D–4G and infected mice by intravenous (i.v.) or i.t. injection. Consistent with previous reports, we observed a level of hepatocyte-tropism after i.v. injection (Figure S3). Through *in vivo* imaging, we found that only the CB promoter could trigger reporter gene luciferase expression in the liver of wild-type mice after i.v. injection (Figure 4D), although liver tissue genome PCR analysis showed that both rAAVs infected liver tissues successfully (Figure 4E). Both the CB and ATM promoters could trigger luciferase expression in subcutaneous tumors after i.t. injection (Figures 4F and 4G). These results suggested that the ATM promoter can efficiently drive target gene expression specifically in tumor cells and thus is a promising promoter for tumor-specific gene therapy.

### rAAV-mediated B8R expression in tumor cells results in tumor elimination and inhibition of lung metastasis in tumor-bearing, VV pre-immunized mice

**Figure 5:**
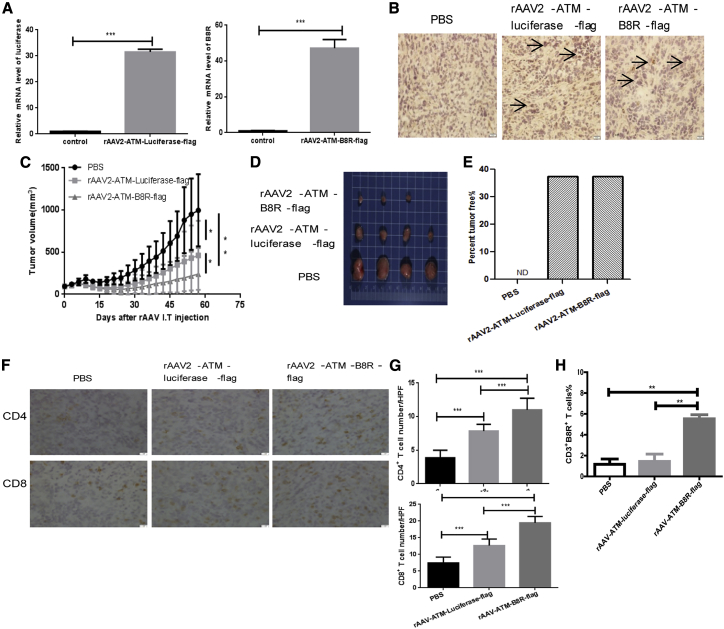
rAAV-mediated B8R expression can delay DT6606 subcutaneous tumor growth in VV pre-immunized mice and promote intra-tumoral immune activation (A) qPCR detection of luciferase and B8R expression in subcutaneous tumors 21 days after PBS or rAAV i.t. injection. Mice used were pre-immunized with VV as described in Figure 1A. Four weeks later, mice were inoculated with 1 × 10^6^ DT6606 cells subcutaneously, and when the tumor volume reached 100 mm^3^, PBS or 1 × 10^11^ vgs of rAAV (expressing luciferase as a control or VV B8R) was injected into each tumor daily for 2 days. n = 3/group, and mean mRNA level ± SEM is shown. (B) IHC of FLAG expression in subcutaneous tumors 21 days after PBS or rAAV i.t. injection is shown. Mice were treated as described in (A). Images are representative of 10 HPFs examined. Magnification 200× is shown. (C) DT6606 subcutaneous tumor growth curve in VV pre-immunized mice after PBS or rAAV i.t. injection is shown; n = 8/group. Tumor growth was monitored twice per week, and tumor volume was calculated. Mean tumor volume ± SEM is shown. (D) Tumors isolated from mice in each group when the tumor volume in PBS group reached 1,500 mm^3^ are shown. Mice were treated as described in (C). (E) The percentage of tumor-free mice in indicated groups was calculated at the experimental endpoint 58 days after rAAV i.t. injection. Mice were treated as described in (C). (F) IHC of tumor-infiltrated CD4^+^ and CD8^+^ T cells in each group 21 days after rAAV i.t. injection is shown. Mice were treated as described in (C). Images are representative of 10 HPFs examined. Magnification 400× is shown. (G) Quantification of CD4^+^ and CD8^+^ T cells in (F) (n = 3/group) from 10 HPFs of each mouse is shown. Mean T cell number ± SEM was shown. (H) Mice were treated as described in (C). Seven days after PBS or rAAV i.t. injection, mice were sacrificed and tumor-infiltrating lymphocytes were examined. CD3^+^B8R^+^ T cells were detected using flow cytometry. n = 3/group. ∗p < 0.05, ∗∗p < 0.01, and ∗∗∗p < 0.001 between the indicated groups of this figure.

We established a DT6606 pancreatic cancer subcutaneous tumor model in immunocompetent mice and pre-immunized the mice with VV 50 days prior to i.t. injection of rAAV-ATM-B8R-FLAG. rAAV-ATM-luciferase-FLAG was used as a control virus. In order to detect whether rAAV can deliver target genes for expression in tumors efficiently, quantitative PCR (qPCR) and immunohistochemistry (IHC) were carried out 21 days after virus injection. Transcription and translation of target genes (luciferase or B8R) were detected in rAAV-infected tumors and not in the PBS control group (Figures 5A and 5B). In the VV pre-immunized mice, tumor growth regressed after treatment with either rAAV-ATM-luciferase-FLAG or rAAV-ATM- B8R-FLAG compared with the PBS group, as both the luciferase protein and the rAAV vector are known to be immunogenic. Importantly, however, the growth rate in the rAAV-ATM-B8R-FLAG-treated group was significantly lower than that of the rAAV-ATM-luciferase-FLAG group (Figure 5C). At the experimental endpoint, the tumor volumes in the rAAV2-ATM-B8R-FLAG treatment group were the smallest among the three groups (Figure 5D). In both the rAAV-ATM-luciferase-FLAG and rAAV-ATM-B8R-FLAG group at the experimental endpoint, 37.5% of mice were tumor free (Figure 5E). Through IHC, we observed more tumor infiltration of CD4^+^ T and CD8^+^ T cells after rAAV infection compared with the PBS group. Importantly, rAAV2-ATM-B8R-FLAG treatment resulted in a statistically greater ability to recruit immune cells compared with PBS and control virus (rAAV-ATM-luciferase-FLAG)-treated groups (Figures 5F and 5G). Using flow cytometry (FC), we observed more infiltration of CD3^+^B8R^+^ T cells in the rAAV-ATM-B8R-FLAG group compared with the PBS and rAAV-ATM-luciferase-FLAG groups (Figure 5H). These results suggested that rAAV-mediated ectopic expression of B8R in DT6606 cells can promote i.t. immune activation, including B8R memory T cells induction, and enhance tumor rejection in a VV pre-immunized DT6606 subcutaneous tumor model.

**Figure 6:**
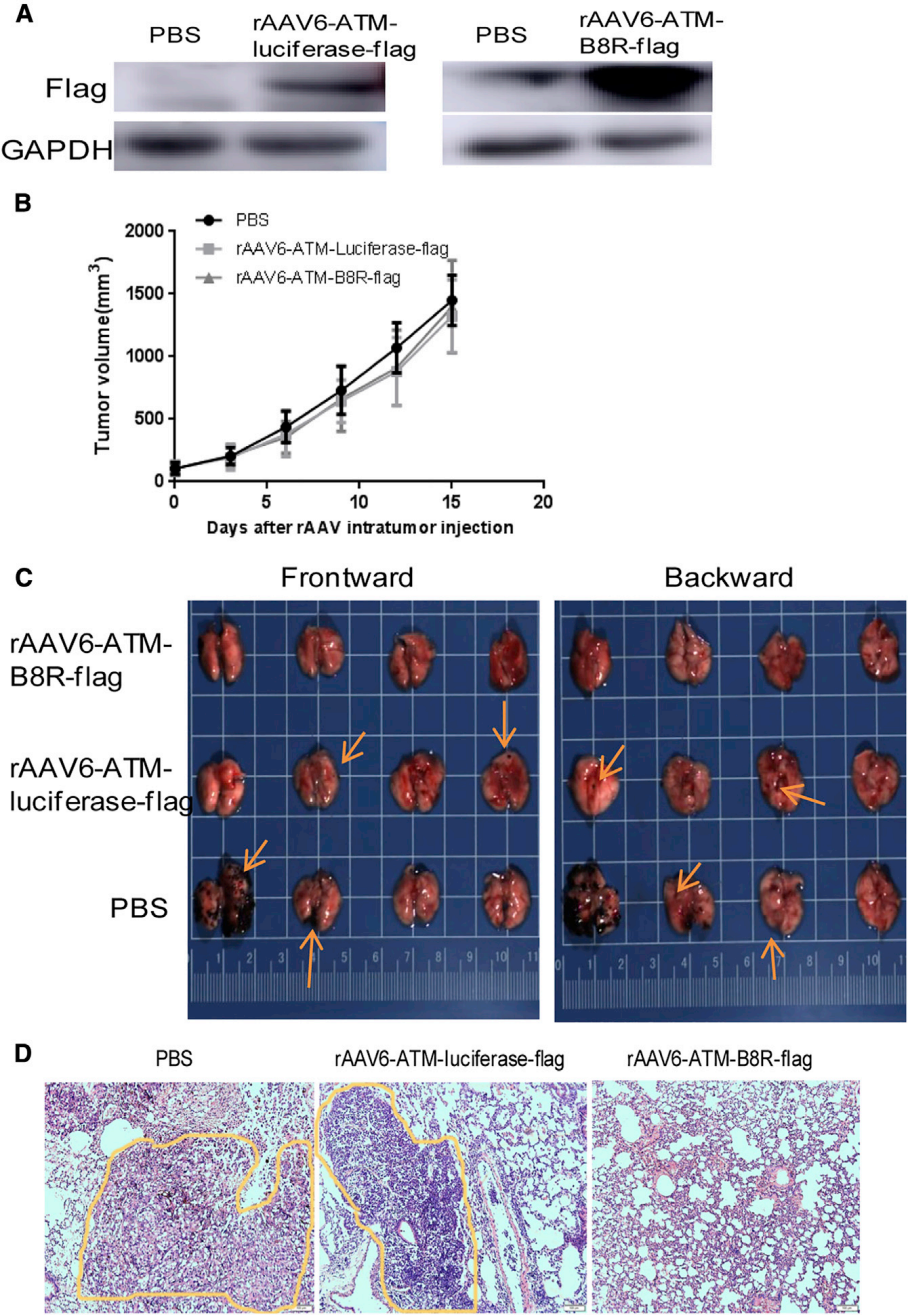
rAAV-mediated delivery of the VV B8R gene to B16 subcutaneous tumors inhibits tumor metastasis in VV pre-immunized mice (A) Expression of luciferase and B8R in B16 subcutaneous tumor 7 days after PBS or rAAV i.t. injection. Mice were pre-immunized as described in Figure 5C. Four weeks after VV immunization, 1 × 10^6^ B16 tumor cells were inoculated in the flank of these mice, and when the tumor volume reached 100 mm^3^, PBS or 1 × 10^11^ vgs of rAAV were injected into each tumor daily for 2 days. (B) B16 subcutaneous tumor growth curve in VV pre-immunized mice after indicated treatment is shown; n = 8/group. Mice were treated as described in (A). Tumor growth was monitored twice per week, and tumor volume was calculated and plotted as mean volume ± SEM. There was no significant difference between different groups. (C) Lung images of sacrificed mice are shown. Mice were treated as described in (A) and sacrificed when the subcutaneous tumor volume reached 1,500 mm^3^. Arrows indicate melanoma metastasis nodules. (D) Representative H&E staining of lungs shows the status of metastatic nodules of cancer in the indicated groups from (C). The yellow circles indicate metastatic nodules. Magnification 100× is shown.

In order to explore the wider application of this strategy, we also detected the antitumor efficacy of rAAV-ATM-B8R gene therapy for treatment of B16 subcutaneous tumors in immunocompetent, pre-immunized mice. i.t. injection of rAAV serotype 6, which is more effective for B16 cell infection *in vitro* (Figure 3A), efficiently triggered target gene expression (luciferase or B8R) in the subcutaneous tumor (Figure 6A). Interestingly, neither vector was effective at retarding tumor growth in this model (Figure 6B) due to the aggressive growth of B16. Strikingly, however, lung metastasis from the B16 tumor was inhibited by the rAAV6-ATM-B8R-FLAG treatment. Zero of eight mice in this group demonstrated lung metastasis, while six of eight mice in the PBS group and five of eight mice in the rAAV6-ATM-luciferase-FLAG group had lung metastasis (Figures 6C and 6D). These results suggested that, although rAAV6-ATM-B8R-FLAG was unable to control primary tumor growth in pre-immunized mice, the remote metastasis in lung could be inhibited by this therapeutic regime. We speculate that the differential effect on primary versus metastases regression may be caused by antigen spreading. The fast growth of B16 tumor cells and low efficiency of AAV infection caused the less effect on primary tumor growth. However, antigen release consequent to the immunogenic cell death of AAV-B8R-infected B16 cells may trigger further identification of B16 tumor cells via different tumor-associated or tumor-specific antigens, which may contribute to metastases regression. We also speculate that a memory pool of CD4^+^ and CD8^+^ T cells may also contribute to metastases regression. However, the semi-liquid condition of the B16 tumor made detection of immune subsets difficult. Using a more solid DT6606 tumor model, we observed that VV pre-immunized mice had the highest number of tumor infiltrating of CD8^+^ effector memory T cells (TEM), but fewer CD8^+^ central memory T cells (TCM) 21 days after the last VV i.t. injection (Figure S4), possibly due to a recruitment of TCM as TEM post-VV treatment.

## Discussion

In this study, we demonstrated that rAAV-ATM-B8R can deliver the VV B8R gene efficiently for specific expression in tumor cells and cause tumor regression in VV pre-immunized mice with subcutaneous pancreatic tumors. Although AAV is widely used in tumor gene therapy,^28^^,^^29^ to our knowledge, this is the first report that AAV gene therapy can be used to activate anti-viral memory T cells to target tumor cells. Our results provide evidence that the novel ATM promoter is tumor specific and has robust expression capability that is similar to the CB promoter both *in vitro* (Figures 4B and 4C) and *in vivo* (Figure 4F). We also found that the human TERT promoter can drive gene expression in mouse cells and tissues, which is consistent with previous report.^30^

As virus-specific memory T cell responses are ubiquitous in populations that were engaged in the smallpox eradication campaign, AAV-mediated virus gene delivery to activate virus-specific memory T cells can be used widely as a strategy for cancer immunotherapy. For translation of this approach into the clinical setting, researchers should firstly analyze the major epitopes of VV that can induce memory T cells in patients. Previous research has identified 25 different HLA-restricted VV epitopes that can induce human CD8^+^ T cells.^31^, ^32^, ^33^ Secondly, it will be important to perform experiments to determine whether all epitopes are valuable in inducing memory T cells. Thirdly, researchers should detect whether there are any target memory T cells in the peripheral blood of patients, as previous research has reported that, although VV-specific memory T cells are long lived in the absence of antigenic re-exposure, they do decline with a half-life of 8–15 years.^34^ If target memory T cells cannot be detected, one approach that could be adopted is immunization of patients with antigens before commencing tumor gene therapy. While here we take advantage of VV memory T cells, those of other viruses, such as influenza virus, Epstein-Barr virus (EBV), severe acute respiratory syndrome coronavirus 2 (SARS-COV-2), or vaccination-induced T-cell-specific epitopes, should also be considered for tumor treatment, as T cell populations in patients are likely. These vaccination strategies may benefit younger generations who have not received the smallpox vaccine.

Although VV also can be used as an immunotherapeutic agent for cancer treatment, there are safety concerns due to unlikely but potential adverse events.^14^ The major advantages of AAV gene therapy are its safety after multiple administrations. Of course, there remain several aspects of this strategy that can be improved. Firstly, i.v. injection of rAAV2 led to liver uptake (Figures 4D, 4E, and S3), which may reduce rAAV2 infection into non-hepatic tumor cells. Hepatocyte tropism is due to expression of a heparin-binding domain in the capsid of AAV2 that promotes its entry into hepatocytes.^27^ This issue may be solved by modification of the capsid protein of AAV through rational design to improve its tumor tropism or by further investigation into the most suitable AAV serotype. Secondly, the rAAV approach was not very effective at treatment of fast-growing tumors, as determined using the B16 subcutaneous tumor model. This is because rAAV cannot replicate in and infect newly divided cells. This issue could be addressed by repeated i.t. injections of rAAV or as a neoadjuvant therapy in combination with surgical removal of the primary tumor after injection. Although rAAV injection could not suppress fast growth carcinoma *in situ* in our model, it could prevent related tumor metastasis or re-challenge effectively (Figures 6 and S2), possibly through antigen-spreading mechanisms. Thirdly, as previous research indicated that memory T cells express immune checkpoint molecules,^35^ combination of rAAV gene therapy with PD-1 inhibitors or other immune checkpoint inhibitors may prevent inactivation of i.t. antitumor T cells and improve the long-term efficacy of tumor treatment. Otherwise, whether this strategy is more effective than utilizing the developed AAV/ATM system for expression of other, none immune-related, therapeutic genes needs further investigation.

In summary, our research confirmed that AAV-mediated delivery of virus gene to redirect pre-existing, virus-specific memory T cells to target tumor is a promising method for cancer treatment.

## Materials and methods

### Cells,plasmids, and viruses

The human embryonic kidney cell line (HEK293) and mouse melanoma cell line (B16) were purchased from ATCC. Human esophageal carcinoma cell lines (KYSE510 and KYSE180) were purchased from DSMZ. Mouse pancreatic cancer cell lines (DT6606 and TB11381) were provided by Prof. David Tuveson (Cancer Research UK Cambridge Research Institute, Cambridge, UK; now at Cold Spring Harbor Laboratory). The murine head and neck squamous cell carcinoma (HNSCC) cell line (SCCVII) was kindly provided by Dr. Osam Mazda (Department of Microbiology, Kyoto Prefectural University of Medicine, Japan). The human esophageal carcinoma cell line (SBRC-EC01) was established in our lab through primary culture from a biopsy of a 57-year-old female esophageal squamous cell carcinoma (ESCC) patient.^36^ The mouse lung cancer cell line (KPL160303S) was derived from *KRas*^*G12D*^*; P53*^*R172H*^; *Ad5-Cre* mice as previously reported.^37^ Cells were all cultured in Dulbecco’s modified Eagle’s medium (DMEM) with 10% fetal bovine serum (FBS) and 100 U/mL penicillin-streptomycin solution at 37°C with 5% CO_2_.

The recombinant VV used in this manuscript was VVLΔTKΔN1L-RFP that has been described previously.^24^ For convenience, this is indicated as VV in the manuscript.

The AAV parental plasmids (pAAV-EGFP/luciferase, pAAV-RC, and pHelper) used in this manuscript were gifts from the lab of Professor Michael J. Passineau in Allegheny Health Network, Highmark.

The lentivirus plasmids (psPAX2, pMD2.G, and FUB-P2A-EGFP-T2A-Puro) used in this manuscript for B8R gene overexpression were purchased from Addgene.

### Establishment of a DT6606 cell line stably over-expressing the B8R gene of VV

The cDNA of B8R was synthesized by Shangya Com (Zhengzhou, China) and cloned into the linearized FUB-P2A-EGFP-T2A-Puro plasmid digested by BamHI (NEB) and EcoRI (NEB) restriction endonucleases. The B8R expression plasmids were packaged into lentiviral particles with the help of psPAX2 and pMD2.G in 293T cells. DT6606 cells cultured in six-well plate were infected by 1-mL viral particles with 8 μg/mL polybrene (Solarbio, Beijing, China) and selected by puromycin to purify B8R stably over-expressed DT6606 cells, indicated as DT6606-B8R in the manuscript. DT6606 cells infected by empty lentiviral particles were used as control cells and indicated as DT6606-control in the manuscript.

### Cell growth curves

Cell proliferation was assessed using IncuCyteZOOM (EssenBioscience). Briefly, DT6606-control and DT6606-B8R cells were seeded in 96-well plates at a density of 5 × 10^4^ cells/well and then cultured in a humidified incubator at 37°C with 5% CO_2_. Cell growth was continuously monitored until the entire well was totally covered by cells and cell growth curve was drawn using GraphPad.

### Differentiation of hiPSCs into hepatocyte cells

Human iPSCs (hiPSCs) derived from the peripheral blood mononuclear cell of a healthy donor,^38^ a gift from Dr. Jianzeng Dong’s lab in the First Affiliated Hospital of Zhengzhou University, were cultured in mTeSR1 medium (STEMCELL; 85,850). For differentiation of hiPSCs to hepatocyte cells, we used Cellartis iPS cell to hepatocyte differentiation kit (Takara) according to the product manual.

### DNA and RNA extraction and real time qPCR

DNA extraction from cells was performed using the Tissue gDNA Isolation kit (Biomega; GD2211-02) in accordance with the manufacturer’s protocol. One thousand nanograms of DNA from each sample was used for agarose gel electrophoresis. RNA was extracted from the cells using Trizol reagent (Thermo Fisher Scientific; 15596026) according to the manufacturer’s instructions. HiScript II 1st Strand cDNA Synthesis Kit (Vazyme; R211-01) was used to reverse transcribe RNA into cDNA according to the manufacturer’s instructions. qPCR was performed using SYBR green (Vazyme), and primers used were CB promoter: forward: 5ʹ-TCCCATAGTAACGCCAATAGG-3ʹ, reverse: 5ʹ-CTTGGCATATGATACACTTGATG-3ʹ; ATM promoter: forward: 5ʹ-CGGTTACGAAGCACTTTCTCG-3ʹ, reverse: 5ʹ-CAGCGGAGAGAGGTCGAATC-3ʹ; EGFP: forward: 5ʹ-GCTACCCCGACCACATGAAG-3ʹ, reverse: 5ʹ-CGGGTCTTGTAGTTGCCGT-3ʹ; β-actin: forward: 5ʹ-CCAGAGGCGTACAGGGATAGCAC-3ʹ, reverse: 5ʹ-TCTCTTCCT CTTGTGCTCTTG-3ʹ. The PCR conditions were as follows: 95°C for 10 min, followed by 40 cycles of 95°C for 15 s and 60°C for 1 min. Samples were processed on an ABI 9700HT system (Applied Biosystems, Foster City, CA). Results were analyzed using SDS 2.2 software, and the relative expression levels of target genes were calculated after normalizing against β-actin.

### IFN-γ ELISA

Mouse spleens were isolated 14 days after VV immunization, mashed through 70 μm BD Falcon cell strainers and flushed with Roswell Park Memorial Institute (RPMI-1640) medium (Sigma Aldrich) containing 10% fetal calf serum (FCS), 1% streptomycin/penicillin, 1% sodium pyruvate, 1% non-essential amino acids (Gibco), and 0.1% β-mercaptoethanol. This medium was called complete T cell medium (CTCM). Splenocytes were lysed in red blood cell (RBC) lysis buffer (Sigma-Aldrich) and re-suspended in CTCM. Suspended cells were co-incubated for 72 h with previously reported VV immunogenic peptides (K3L, B2R, B8R, A8R, and A3L)^25^ separately, and mesothelin peptide was used as control. IFN-γ production was assessed by ELISA (eBioscience) according to manufacturer’s protocol. The sequences of immunogenic peptides used in this experiment are K3L: YSLPNAGDVI; B2R: YSQVNKRYI; B8R: TSYKFESV; A8R: ITYRFYLI; and A3L: KSYNYMLL. All these peptides were synthesized by GL Biochem (Shanghai, China).

### Flow cytometry (FC) analysis

Splenocytes or tumor-infiltrating lymphocytes (TILs) were extracted from mice and diluted in CTCM. Cells were pushed through a 70-μm cell strainer to create a single-cell suspension, centrifuged, and the pellet was re-suspended in 5 mL red blood cell lysis buffer (Sigma-Aldrich) for 5 min. We prepared at least 1 × 10^6^ lymphocytes for each staining and the staining antibodies used were: anti-mouse CD3e-fluorescein isothiocyanate (FITC) (eBioscience; 11-0031-86); CD4-antigen-presenting cell (APC) (eBioscience; 17-0041-82); CD8a-APC (eBioscience; 12-0081-85); and B8R pentamer-PE (customized from Proimmune). After 1 h incubation at room temperature, cells were acquired on an FC scanner, and data were analyzed using FlowJo software (Tree Star).

### Immunohistochemistry

Tissues collected at different time points were processed and stained by H&E or immunohistochemistry (IHC) as previously described.^39^ For IHC, consecutive 6-μm-thick slices from each sample were de-paraffinized in dimethyl benzene, rehydrated through a graded ethanol series, and incubated with fresh 3% H_2_O_2_ for 10 min to quench endogenous peroxidase activity. After a rinse in phosphate-buffered saline (PBS), antigen retrieval was performed by microwave heating. Following incubation in 10 mmol/L citrate buffer (pH 6.0) for 20 min, sections were incubated with primary antibodies for CD4 (BioLegend; 1:500), CD8 (BioLegend; 1:500), FLAG (Abcam; 1:500), or VV (Invitrogen; 1:500) at 4°C overnight. The corresponding secondary antibody (Dako) was added for incubation at 37°C for 30 min before reaction with diaminobenzidine and counterstaining with hematoxylin. Images were captured using a Leica IM50 40× microscope (Imagic Bild Verarbeitung AG, Wetzlar, Germany). The CD4- and CD8-positive cell number was calculated by two researchers who were blinded to the experimental grouping, and 10–15 microscope fields at magnification 400× were randomly chosen in each slide to calculate the average positive cells number per high power field (HPF).

### Western blot

Western blot analysis was performed according to protocols previously described.^40^ Briefly, cells were washed twice with cold PBS and scraped in RIPA lysis buffer (150 mM NaCl, 1% NP-40, 0.5% deoxycholic acid, 0.1% SDS, and 50 mM Tris [pH 8.0]) supplemented with 0.1 mM phenyl methyl sulfonyl fluoride (PMSF). Cell lysates were incubated on ice for 30 min and centrifuged at 15,000 × *g*, 4°C for 15 min. Proteins in the supernatant were extracted and quantified using bicinchoninic acid (BCA) protein assay (Pierce, Rockford, IL). Cell lysates were loaded with 4× loading dye (Tris-HCl [pH 7.4], 1% SDS, glycerol, dithiothreitol, and bromophenol blue) and subjected to electrophoresis on 8% or 10% SDS-polyacrylamide gels and then transferred onto nitrocellulose membrane (Bio-Rad, Richmond, CA, USA). The membrane was blocked with 5% milk in Tris-buffered saline (TBS) with Tween 20 and incubated with primary antibodies (anti-FLAG [1:1,000] [Abcam, ab205606], anti-GAPDH [1:5,000] [Proteintech, 60004-1]). After washing with TBS supplemented with 0.05% Tween 20 for 30 min, secondary antibody conjugated to horseradish peroxidase (Jackson Immunoresearch Laboratories, WestGrove, PA, USA) was added to the membrane for visualization.

### Construction of recombinant adeno-associated virus vectors

To generate an AAV vector with the ATM promoter, pAAV-CB-EGFP/luciferase vector, a gift from lab of Professor Michael J. Passineau, was used as a template plasmid. Firstly, the ATM promoter was synthesized with Mlu-I and Age-I restriction sites by Sangon Biotech, resulting in Mlu-I-ATM promoter-Age-I sequence; the sequence of ATM promoter is showed in Figure 3B. The AAV-CB-EGFP/luciferase vector was linearized by Mlu-I and Age-I and used as backbone vector. The Mlu-I-ATM promoter-Age-I sequence was ligated into the backbone vector, and the targeting vector pAAV-ATM-EGFP/luciferase was generated.

To generate the AAV-ATM-B8R vector, the cDNA of the B8R gene from Lister VV strain was inserted into the rAAV vector by homologous recombination. The pAAV-ATM-EGFP vector was used as template, the EGFP gene was deleted by NcoI and HindIII digestion, and the B8R-FLAG sequence was inserted into this position. The B8R gene fragment was amplified by PCR using the genome of DT6606-B8R cell as template, and the FLAG coding sequence was added to the amplified product. The primer sequences were forward: 5ʹ-GTACCGAGGCACCGGTCGCCACCGGATCCATGAGATATATTATAATT-3ʹ; reverse: 5ʹ-TCGATAAGCTTACCGGTTTACTTGTCATCGTCGTCCTTGTAGTCTGAATATTTAGTCAA-3ʹ. The digestion product and PCR product were ligated by homologous recombination to obtain pAAV-ATM-B8R-FLAG. pAAV-ATM-Luciferase was used as a template to construct pAAV-ATM-Luciferase-FLAG through homologous recombination. The primer sequences were forward: 5ʹ-CGAGGCACCGGTCGCCACCATGGAAGACGCCAAAAACATA-3ʹ; reverse: 5ʹ-ACGGTATCGATAAGCTTTTACTTGTCATCGTCGTCCTTGTAGTCCACGGCGATCTTTCC-3ʹ.

### Production of rAAV

rAAV was produced using the triple-transfection method.^41^ Three plasmids (pAAV-EGFP/luciferase, pAAV-RC, and pHelper) were transfected into HEK293 cells by polyethylenimine (PEI) (Sigma; 91902). For each 15 cm^2^ cell culture plate, plasmids (62.4 μg) with PEI (plasmid: PEI = 1:1) were added to the culture plate. The appropriate plasmid used for transfection was calculated according to the size of plasmid fragment. Six hours after transfection, the medium was discarded and replaced with fresh medium. Seventy-two hours later, the cells were collected by centrifugation, and the cell pellet was re-suspended with 1 mL rAAV lysis buffer (15 mL of 5 M NaCl and 25 mL of 1 M Tris HCl [pH 8.5]) added to 500 mL ultra-pure water) per plate. After three freeze-thaw cycles, 1 μL benzonase (Sigma; E1014-250KU) was added before incubating at 37°C for 40 min. Then, virus was subjected to iodixanol gradient centrifugation, 40,000 rpm for 18 h at 18°C. The purified virus was collected and stored in −80°C. The total vector genome number was determined by qPCR.

### Infection of cells by rAAV

Cells were seeded in 24-well plates and incubated for 24 h in 37°C incubator with 5% CO_2_. The medium was removed and replaced with 0.3 mL of fresh medium and appropriate amount of recombinant virus (rAAV-CB-GFP; MOI = 10^5^) was added to each well. After a 10-h incubation, the medium was removed and cells were further incubated for 60 h in fresh complete medium. Cells were digested and collected in cytometer tubes for FC analysis by ectopically expressed GFP. Data were analyzed using FlowJo software. Infection efficiencies were quantified by means of percentage of EGFP-positive cells and the average intensity of fluorescence per cell.

### Animal experiments

Male C57/Bl6 mice were purchased from Beijing Vital River Laboratory Animal Technology and housed in the animal facilities of Zhengzhou University with 12 h light and 12 h dark cycles and free access to water and food. All animal experiments were carried out under the terms of the mouse welfare and ethics of Zhengzhou University.

For evaluation of the tumor-specific expression of the ATM promoter, 4- to 6-week-old mice were injected with 1 × 10^11^ copies of viral genome (vg). AAV-CB-luciferase or AAV-ATM-luciferase in 100 μL PBS was injected i.v. or i.t. All mice recovered from the injection quickly without loss of mobility or interruption of grooming activity.

For the VV pre-immunized subcutaneous tumor model, 5- to 6-week-old mice were pre-immunized by injection of 5 × 10^6^ plaque-forming units (PFUs) VV/mouse i.m. twice with a 2-week interval. Four weeks after the second immunization, mice were subcutaneously inoculated with 1 × 10^6^ of B16, DT6606, DT6606-B8R, or DT6606-control tumor cells. For evaluation of the therapeutic effect of VV, 1 × 10^8^ PFUs VV/mouse were administered daily for 5 days, through multi-spot i.t. injection when the subcutaneous tumor volume reached 80–100 mm^3^. For evaluation of the therapeutic effect of rAAV, 1 × 10^11^ vg rAAV/mouse was administered for 2 consecutive days through multi-spot i.t. injection. The mice were divided into three groups: PBS group; AAV-ATM-Luciferase-FLAG group; and AAV-ATM-B8R-FLAG group. The length and width of the subcutaneous tumor were measured every 3 days with a vernier caliper, and tumor volume was calculated according to the equation volume = (length × width × width)/2 × 3.14.

For animal *in vivo* imaging, mice were anesthetized with 2% isoflurane and oxygen. D-luciferin, potassium salt was used as a substrate and injected intra-peritoneally (i.p.) at a dose of 150 mg/kg body weight. For each mouse, the images were taken by PerkinElmer IVIS spectrum machine, 10–15 min after substrate injection.

### Statistical analysis

Statistical analysis was carried out using GraphPad Prism 5 and SPSS 19.0 software. The results were represented as mean ± standard error of the mean (SEM). Differences were analyzed using the Student’s t test to compare significance between two groups and one-way ANOVA test to compare significance between more than two groups or Kaplan-Meier survival analysis. Differences were considered statistically significant when the p < 0.05.

## Supplemental information

Document S1. Supplemental methods and Figures S1–S4

Document S2. Article plus supplemental information
