# Supplementary material for: Redirecting anti-Vaccinia virus T cell immunity for cancer treatment by AAV-mediated delivery of the VV B8R gene
Source: Mol Ther Oncolytics. 2022 Apr 25;25:264–75. doi: 10.1016/j.omto.2022.04.008 (PMC9114156; doi:10.1016/j.omto.2022.04.008)
Supplement: Document S1. Supplemental methods and Figures S1–S4 [file mmc1.pdf]

OMTO, Volume *25*

Supplemental information

Redirecting anti-Vaccinia virus T cell immunity for cancer treatment by AAV-mediated delivery of the VV B8R gene

Dujuan Cao, Qianqian Song, Junqi Li, Louisa S. Chard Dunmall, Yuanyuan Jiang, Bin Qin, Jianyao Wang, Haoran Guo, Zhenguo Cheng, Zhimin Wang, Nicholas R. Lemoine, Shuangshuang Lu, and Yaohe Wang

Supplemental methods:

***In vitro* splenocytes stimulation and cell cytotoxicity**

Murine spleens were isolated 7 days after treatment, mashed through 70µm BD Falcon™ cell strainers and flushed through with complete T-cell media (Roswell Park Memorial Institute (RPMI)-medium 1640 (Sigma Aldrich), 10% FCS, 1% streptomycin/ penicillin, 1% sodium pyruvate and 1% non-essential amino acids (Gibco®) and 0.1% β-mercaptoethanol). Splenocytes were re-suspended in red blood cell (RBC) lysis buffer (Sigma-Aldrich) and re-suspended in complete T-cell medium.

Then cell suspensions were incubated for 72 hours with B8R peptides or heat inactivated VV (60°C water bath for 10min) prior to assessment of cell cytotoxicity by LDH cytotoxicity assay kit from Promega according to the user manual.

Supplemental Figures and Figure legends:

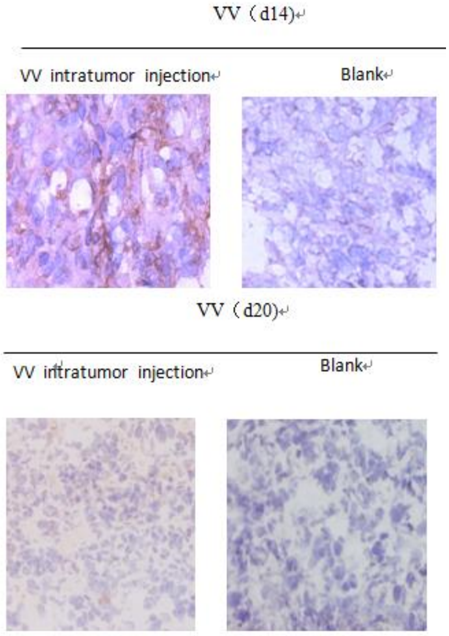

Supplemental Figure 1. Immunohistochemistry identifying VV in DT6606 tumor sections 14 days or 20 days after last VV I.T injection. 1x10^8 PFU VV/mouse was administered through I.T injection daily for 5 consecutive days. 14days (d14) or 20days (d20) after last VV I.T injection, mice were sacrificed and tumor tissues were collected for IHC using an antibody to detect VV.

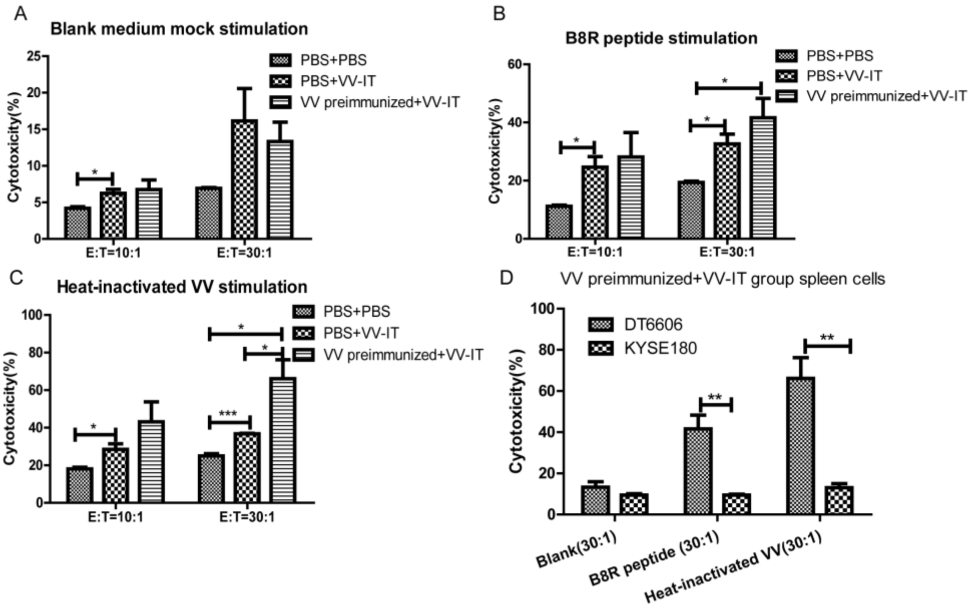

Supplemental Figure 2. B8R peptide or heat-inactivated VV stimulated spleen cells can kill B8R-negative DT6606 cells efficiently, but not unrelated KYSE180 cells. For pre-immunization, C57/Bl6 mice were immunized using 100µl PBS or VV at a MOI of 5x10^6 PFU/mouse through intramuscular injection twice with a 2 week interval. 4 weeks after VV or mock immunization, 1x10^6 DT6606 cells were inoculated into the flanks of the mice. 10 days later, once the volume of the tumor reached 100mm^3,

100μl PBS or $1x10^8$ PFU VV/mouse was administered through I.T injection daily for 5 consecutive days. 7 days after the last injection of VV or PBS, mice were sacrificed and spleens extracted. Splenocytes were stimulated by blank medium (**A**), B8R peptide (**B**) or heat-inactivated VV (**C**) for 72h before incubation with DT6606 cells (**A, B, C, D**) or KYSE180 cells (**D**) at the indicated ratios for 4 h. E:T means effector cells (spleen cells) : target cells (DT6606 or KYSE180) ratio. The cell cytotoxicity was detected by LDH assay. **$p<0.05$, **$p<0.01$, ***$p<0.001$.*

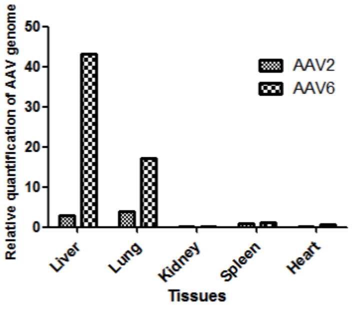

Supplemental Figure 3. Q-PCR detection of AAV genomes in different organs of mouse seven days after intravenous injection of AAV. Mice were infected by AAV2 or AAV6 at a MOI of $1x10^{11}$ vg/mouse through tail vein injections once.

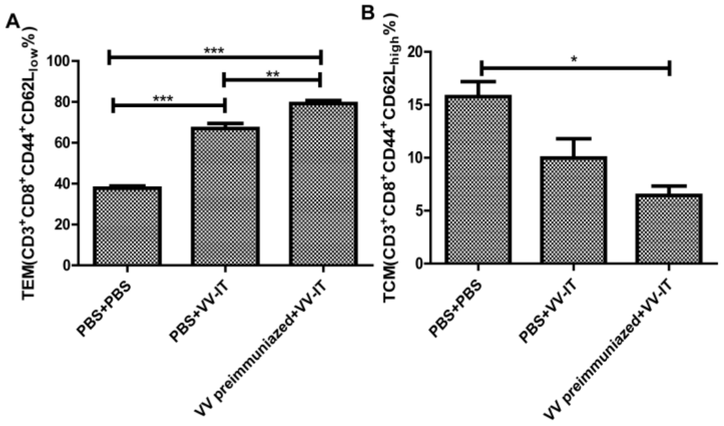

Supplemental Figure 4. Quantification of effector memory (TEM) and central memory (TCM) $CD8^+$ T cells in the DT6606 tumor after indicated treatment. For pre-immunization, C57/Bl6 mice were immunized using 100μl PBS or 100μl VV at a MOI of $5x10^6$ PFU/mouse through intramuscular injection twice with a 2 week interval. 4 weeks after VV or mock immunization, $1x10^6$ DT6606 cells were inoculated into the flanks of the mice. About 10 days later, once the volume of the tumor reached to about $100mm^3$, 100μl PBS or $1x10^8$ PFU VV/mouse was administered through I.T injection daily for 5 consecutive days. 21 days after the last injection of VV or PBS, we sacrificed the mice and examined the tumor infiltrating T cells using flow cytometry to detect the TEM (**A**) and TCM (**B**) cells. **$p<0.05$, **$p<0.01$, ***$p<0.001$.*
